# Design, modeling, and preliminary evaluation of a 3D-printed wrist–hand grasping orthosis for stroke survivors

**DOI:** 10.1017/wtc.2024.18

**Published:** 2024-11-08

**Authors:** Elissa D. Ledoux, Eric J. Barth

**Affiliations:** 1 Department of Mechanical Engineering, Vanderbilt University, Nashville, TN, USA; 2 Department of Engineering Technology, Middle Tennessee State University, Murfreesboro, TN, USA

**Keywords:** soft wearable robotics, rehabilitation robotics, design

## Abstract

Stroke causes neurological and physical impairment in millions of people around the world every year. To better comprehend the upper-limb needs and challenges stroke survivors face and the issues associated with existing technology and formulate ideas for a technological solution, the authors conversed with 153 members of the ecosystem (60 neuro patients, 30 caregivers, and 63 medical providers). Patients fell into two populations depending on their upper-limb impairment: spastic (stiff, clenched hands) and flaccid (limp hands). For this work, the authors chose to focus on the second category and developed a set of design constraints based on the information collected through customer discovery. With these in mind, they designed and prototyped a 3D-printed powered wrist–hand grasping orthosis (exoskeleton) to aid in recovery. The orthosis is easily custom-sized based on two parameters and derived anatomical relationships. The researchers tested the prototype on a survivor of stroke and modeled the kinematic behavior of the orthosis with and without load. The prototype neared or exceeded the target design constraints and was able to grasp objects consistently and stably, as well as exercise the patients’ hands. In particular, donning time was only 42 s, as compared to the next fastest time of 3 min reported in literature. This device has the potential for effective neurorehabilitation in a home setting, and it lays the foundation for clinical trials and further device development.

## 1. Introduction

Stroke affects millions of people across the globe every year, particularly the elderly, and accounts for roughly three-quarters of the neurologically impaired population (Ledoux, 2024). During a stroke, a part of the brain dies and loses connection to the rest of the body. For many, this results in upper-limb impairment, such as lack of hand control, including difficulties grasping and opening, instability, reduced sensation (Hospitals, 2024), and loss of fine and gross motor skills (Eschmann et al., 2019). Survivors of such a debilitating, life-changing event are often rendered dependent on others for even the most basic tasks. If left unaddressed, their struggles compound over time, resulting in fatigue, frustration, and a gamut of other issues. Compounding their physical impairment, survivors often suffer related mental and financial hardships. As a result, patients need a low-cost and easy-to-use device meeting their basic needs for gross motor skills recovery.

As explained by therapists and patients in our customer discovery conversations, the limitations of current technology abound: physical rigidity, operation procedure, sizing, and/or cost. Passive devices are inexpensive compared to powered ones, but they lack the ability to actively move the patient’s hand. They either hold the hand in a static position (static splints) or cause the hand to move unnaturally by coupling wrist motion to finger motion (linkage based). While highly refined and technologically advanced, the powered devices, such as the Myomo, Bioness, GloreHa, EnableMe, and Syrebo (EnableMe, 2015; BTL Robotics, 2024; Bioness, 2024; Myomo, 2021; Syrebo, 2023), are in many cases inaccessible to patients at home due to cost or other factors. The EnableMe and GloreHa are intended for use in a clinical setting with a virtual reality station (EnableMe, 2015; BTL Robotics, 2024). While these can be powerful rehabilitation tools, they are not accessible to patients at home for daily assistance. While statistics on cost and weight for most of these powered devices are not publicized, the Myomo is advertised as weighing approximately 2 kg (Myomo, 2021), more than twice the weight of most research prototypes, and could be difficult for someone with neurological impairment to don and maneuver. The Bioness costs approximately 500 USD (Bioness, 2024), which patients already experiencing financial constraints may find difficult to afford. The Syrebo is the lowest profile, lightest, and presumably the most comfortable of these devices due to its soft structure compared to the others; however, it is similar in cost to the Bioness and can be difficult for patients to don independently due to its individual finger compartments (requiring fine motor skills) (Syrebo, 2023). Neurologically impaired patients need access to low-cost, lightweight, user-friendly assistive devices intended for at-home use, in order to enable daily rehabilitation and facilitate their journey to independence.

Although the approach to exoskeletons and orthoses has traditionally involved rigid links with electromechanical actuation (Johnson et al., 2001; Ueki et al., 2012; Ates et al., 2015; Gasser et al., 2017), research in this area has shifted toward soft robotics (Rahman et al., 2000; Kadowaki et al., 2011; Polygerinos et al., 2015; Biggar and Yao, 2016; Zhao et al., 2016; Saharan et al., 2017; Yap et al., 2017a, 2017b; Portnova et al., 2018; J. Wang et al., 2019; Yoo et al., 2019; Zhou et al., 2019; Flechtner et al., 2020; Haghshenas-Jaryani et al., 2020; Hong et al., 2020; L. Wang et al., 2020; Yurkewich et al., 2020; Shi et al., 2021; Guo et al., 2022; Kim et al., 2022; Kladovasilakis et al., 2022; Rieger and Desai, 2022; Suulker et al., 2022; Tran et al., 2022; Lai et al., 2023; Li et al., 2023) over the last decade. Soft robotics are often ideal for rehabilitation due to their novel materials and human-friendly interaction, compared to rigid robotics.

In recent years, many wrist–hand orthoses (WHOs) have been developed in research, most of them (Ueki et al., 2012; Kadowaki et al., 2011; Polygerinos et al., 2015; Biggar and Yao, 2016; Zhao et al., 2016; Gasser et al., 2017; Saharan et al., 2017; Yap et al., 2017b; J. Wang et al., 2019; Yoo et al., 2019; Zhou et al., 2019; Hong et al., 2020; L. Wang et al., 2020; Yurkewich et al., 2020; Guo et al., 2022; Kim et al., 2022; Kladovasilakis et al., 2022; Suulker et al., 2022; Tran et al., 2022; Lai et al., 2023) for purely assistive purposes. Very few research prototypes have both therapeutic and assistive purposes (Ates et al., 2015; Yap et al., 2017a; Haghshenas-Jaryani et al., 2020; Shi et al., 2021). All of these come in limited sizing and are difficult for subjects to don (independently or not), due to their defining characteristic of individual finger compartments, an obstacle for any patient lacking volitional hand control. There is a clear need for an effective multipurpose assistive/therapeutic WHO that can fit a large range of patients appropriately and is easy to don and doff.

The authors’ aim is to develop an easily custom-sized WHO for survivors of stroke, to provide both power grasping assistance and therapeutic manual exercise in order to expedite their journey to independence. The motivation for this device, and reasoning behind its form and functions, stems from the customer discovery conversations conducted with the neurologically impaired population. While stroke survivors are the initial target demographic, this device has the potential to benefit other neurologically impaired patients as well. The primary goal is to assist patients with stable power grasps of common household objects, for performing activities of daily living (ADLs). The secondary goal is to provide a therapy tool for exercising the hand and rebuilding the mind–body connection. Testing the assistive benefits of the prototype was focused on more heavily in this study, and while therapeutic benefits are predicted with repeated use of the device, they are only quantifiable over a longer period of time than the scope of this work entails. The orthosis prototype subsequently described and evaluated in this feasibility assessment has been designed using criteria based on the verbalized desires of stroke survivors, caregivers, and medical providers. Because of this, the device involves a design shift from the typical glove style of soft robotics to a single-sided, easily custom-sized, inflatable mitten and directly addresses patients’ medical needs.

## 2. Custom sizing modeling

One limitation of both commercially available orthoses and most existing research prototypes is limited custom sizing. For these, the only size adjustment (if any) available is by tightening straps. The one exception, to the authors’ knowledge, is a single 3D-printed, passive, rigid hand orthosis design (Portnova et al., 2018), for which size modifications are possible if the manufacturer performs certain mathematical calculations and modifies nine variables. While soft robotics are friendlier to size variation in hands due to continuous dynamics, one size does not fit all. Patients’ hand sizes vary depending on age, gender, muscle tone, blood circulation, and hereditary factors. An incorrectly sized orthosis could either prevent motion or constrain the hand to move unnaturally, as well as causing discomfort, abrasion, slipping, and/or grasp impediment. For these reasons, the authors chose to develop mathematical models characterizing hand anatomy for ease of custom sizing the orthosis design. Advantages of the authors’ approach over the one in Portnova et al. (2018) are as follows: (1) simplification using fewer design parameters and eliminating the need for the manufacturer to perform calculations, (2) using an open source CAD platform (Onshape), and (3) including effective powered actuation.

By assessing anthropometric data and relationships between different anatomical parameters on the hand and wrist, it is possible to relate some segment lengths to others. Because some anatomical dimensions can then be calculated from others, the set of required measurements reduces. These measurements then become the set of critical design parameters.

**Figure 1.**
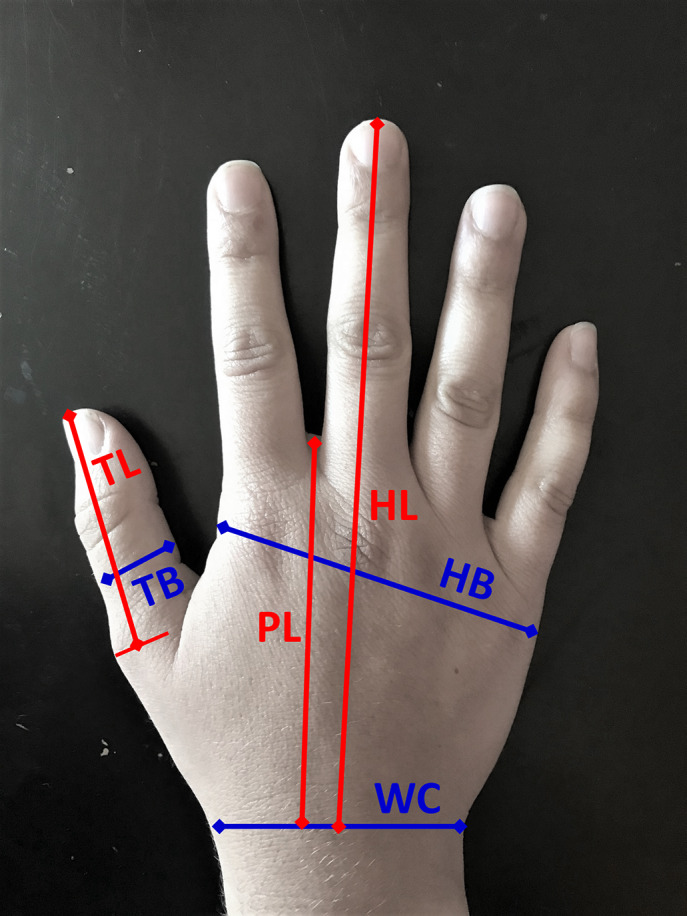
Anatomical measurements.

Using anatomical variables from a US military assessment (Gordon et al., 1989), the authors analyzed and compared the data in Dreyfuss (1967) and Gordon et al. (1989) for adult males every five percentile points from 5 to 95. The six specific variables chosen for the analysis are listed in Table 1 and shown in Figure 1 on a hand.

**Table 1.** Anatomical term

Abbreviations	Anatomical terms
HB	Hand breadth
HL	Hand length
PL	Palm length
TB	Thumb breadth
TL	Thumb length
WC	Wrist circumference

The analysis checked for ratio relationships between the variables. Each variable was plotted against the others, and a best-fit line passing through the origin was found for each relationship:
(2.1)

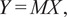

where *Y* represents the dependent variable, *X* represents the independent variable, and the ratio relationship *M* is the slope of each line. The relationship was assumed to be valid if the correlation coefficient 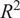 > 0.98. Several valid ratio relationships were found for the anatomical data. Hand length (HL) correlated strongly to wrist circumference (WC), palm length (PL), hand breadth (HB), thumb breadth (TB), and thumb length (TL), and many of these variables correlated to each other as well.

**Figure 2.**
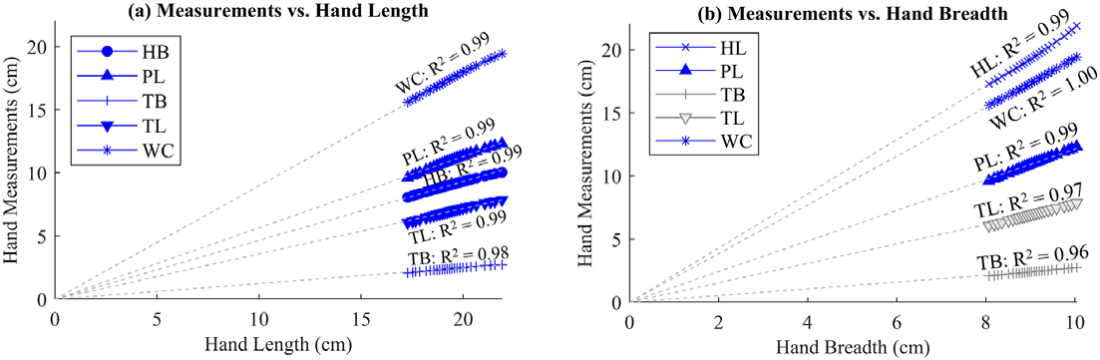
Hand/wrist measurement relationships. (a) Male hand length (Gordon et al., 1989) is plotted on the independent axis, and the other measurements (Dreyfuss, 1967; Gordon et al., 1989) are on the dependent axis. (b) Male hand breadth (Gordon et al., 1989) is plotted on the independent axis, and the other measurements (Dreyfuss, 1967; Gordon et al., 1989) are on the dependent axis. Blue markers represent valid ratio relationships, gray markers represent invalid ratios, and the best-fit lines are light gray dashes.

Sample plots showing the two variables that correlate most strongly with others (HL) and one that does not (TB) with their respective relationships are in Figure 2a,b, respectively. The variable in question is plotted on the independent axis, while the other measurements are plotted on the dependent axis, and best-fit lines provide a visual point of comparison.

The valid relationships were then assessed to determine the feasibility of eliminating certain measurements and simply calculating them from others, reducing the number of parameters required for design. Each design dimension for the orthosis CAD was then based on a ratio relationship to a single input dimension. Any required dimensions that were unable to be related to others by a ratio (e.g., wall thickness, strap width) were input directly as constants.

After comparing all data, a critical parameter set was determined to consist of two variables, one length (HL) and one width (HB), reducing the number of measurements required from six to two. Although mathematically, the data indicate that using just HL would be sufficient, the authors chose to use both a length and a width measurement to better accommodate potential size variability of other ethnicities and gender not included in the available dataset. The independent variables X (input dimensions) are HB and HL. The dependent variables Y (design dimensions) are PL, TB, TL, and WC. The final ratios used in the orthosis CAD design are tabulated in Table 2. The data columns are *X* (independent variable), *Y* (dependent variable), 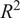 (correlation coefficient), and *M* (relationship ratio of dependent to independent variable).

**Table 2.** Anatomical ratios

*X*	*Y*	*R* ^2^	*M*
HB	WC	0.999	1.928
HL	PL	0.993	0.565
HL	TL	0.987	0.359
HL	TB	0.981	0.125

The size of the orthosis can be easily adjusted by changing the two design parameter variable values, HB and HL. The files for 3D printing accommodate sizes ranging from 10th percentile female to 85th percentile male.

## 3. Orthosis design

To better understand the upper-limb needs and challenges survivors of stroke and other neurological events face and to determine a target end user, the authors conducted customer discovery conversations with 153 people in the ecosystem (60 patients, 30 caregivers, and 63 medical providers) as part of the National Science Foundation I-Corps Program. These customer discovery conversations were informal discussions that sought to understand (a) what upper-limb problems stroke survivors experience and (b) the magnitude and frequency of those pain points, in order to identify addressable needs of the neurologically impaired population and help formulate ideas for a potential technological solution. See the Supplementary Material for a list of discussion points. Patients and caregivers provided personal perspectives based on individual experiences, while doctors, nurses, and physical and occupational therapists shared information that is common knowledge to the medical community but unfamiliar in the engineering domain. The customer discovery revealed problems such as reduced sensation and volitional control in the affected limb and extreme weakness, difficulties that are often compounded by cognitive impairment and financial hardship. The team also learned that patients fell into two populations depending on their upper-limb impairment: spastic/toned (stiff, clenched hands) and flaccid (limp hands). For the subsequently described body of work, the research team chose to focus on a technological solution for the second category of patients (the first category is addressed in a separate work; Ledoux et al., 2024). This group struggles to use their hands due to flaccidity and needs basic assistance to open and close their hands for power grasping while performing ADLs, as well as simply for exercise to retrain mind–body coordination and prevent edema (Cramer, 2018). Based on the information collected from customer discovery, the authors derived a set of design constraints and then developed a powered wrist–hand grasping orthosis to aid in recovery. The set of design constraints derived from customer discovery are as follows:

1. *Easy to don and doff.* Since the patients lack motor skills (Eschmann et al., 2019), ease of donning is essential. They are unable to control their fingers and must manually push them into position, making individual finger motion practically impossible and often requiring the help of a caregiver in a procedure that can last several minutes. Ideally, a patient could single-handedly put the orthosis on their hand, or remove it, in less than one minute.
2. *Flexible and comfortable.* Many patients complained that their static splints are painful due to the constant aggressive stretch, so they need something comfortable, or they will not wear it. And since a common side effect of neurological impairment is reduced sensation in the affected limb (Hospitals, 2024), preventing abrasion is critical to prevent skin damage and infection.
3. *Grasping assistance.* Due to the extreme weakness that many stroke survivors face, an orthosis should assist the hand with stable power grasps. Many patients yearn to be able to use the affected hand as a supporting member for bimanual tasks, so the affected hand could hold an object stable while a more dexterous motion is performed with the unaffected hand. Often, patients learn compensatory movements that render the affected hand unnecessary, thereby making the possibility of rebuilding the connection between the brain and the affected limb more remote as time progresses. Use of the affected hand is both desired by patients and perhaps relevant to regaining functionality. The grip force target is 40 N to reach the threshold for ADLs (Riddle et al., 2018).
4. *Therapeutic exercise.* To prevent contractures and edema and the hypothesized rebuilding of the mind–body connections, patients’ hands need to be exercised multiple times a day. Due to patients’ lack of volitional control, the orthosis should open and close to cyclically exercise the hand, reducing the required time and effort by therapists and caregivers and enabling more therapy in a given time frame.
5. *Intelligent:* Intuitive control and sensory feedback – any device for a neurologically impaired person must be easy to use and safe. Powering and controlling the device should be simple, and force or pressure monitoring should prevent over-extension of joints.
6. *Affordable.* As most stroke survivors are no longer able to maintain a full-time job and often require caregiver assistance, they also face financial challenges. A device should be as low cost as possible so they can afford it, ideally below 100 USD.
7. *Portable:* Lightweight and untethered – in order for a patient to easily transport and use a device, it must be unchained from external equipment. Furthermore, the proportion of weight on the distal limb should be minimized (ideally <0.5 kg) in order to reduce the required torque to lift the arm and hand, with heavier components located more proximally.

For this prototype, the authors chose to prioritize the first four design constraints (pertaining to the end effector): easy to don and doff, flexible and comfortable, assistive grasping, and therapeutic exercise. The other design constraints (pertaining to the full system), intelligent, affordable, and portable, were considered secondary priorities and will be addressed in future work as the project develops.

**Figure 3.**
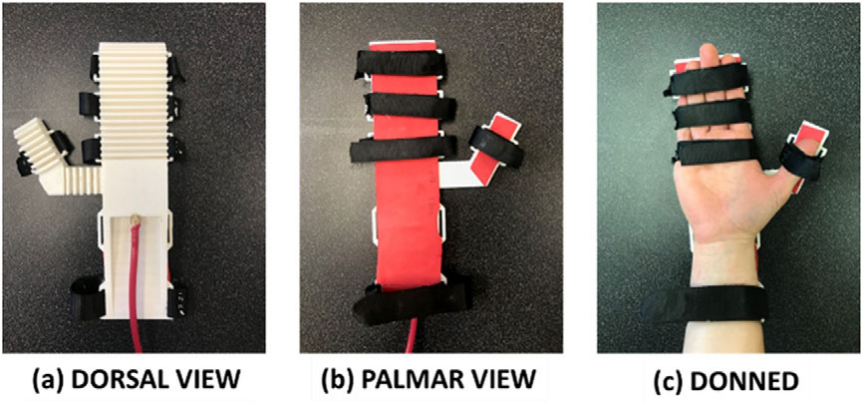
Orthosis prototype. The photographs, clockwise from top left, show the prototype with (a) dorsal view, (b) palmar view, and (c) palmar donned.

The orthosis prototype, shown in Figure 3, uses a single-sided mitten concept with unidirectional fluid-powered actuation and wrist support. The actuator, which fits on the dorsal side of the hand, is 3D-printed from TPU of Shore 85A hardness. The fingers and thumb have hollow trapezoidal bellows with arches in the base for air flow. These bellows inflate to curl the actuator for grasping. The shape of the thumb is angled at first 90° and then 30° from the fingers, parameters determined experimentally for adequate power grasp shape. The phalanges are attached to a solid palm with a few internal channels for air flow. The wrist tapers down from the palm for support and comfort. Foam padding is adhered to the underside of the actuator for comfort and to prevent abrasion. The orthosis fastens to the patient’s hand and wrist by five straps. A 1-inch-thick, adjustable nylon strap with hook and loop fasteners secures the wrist support to the forearm, while the fingers and thumb use 0.75-inch-thick polyester straps for adjustment and security. The straps are low profile and strategically placed so as to preserve palmar sensation for grasping and allow thumb opposition. The entire design is low cost and dimensioned based on ratios of HL and HB (as explained in Section 2; Custom Sizing Modeling), except for a few constants such as overall length (limited by printer bed size) and strap width. The 50th percentile male size orthosis weighs 276 g.

**Figure 4.**
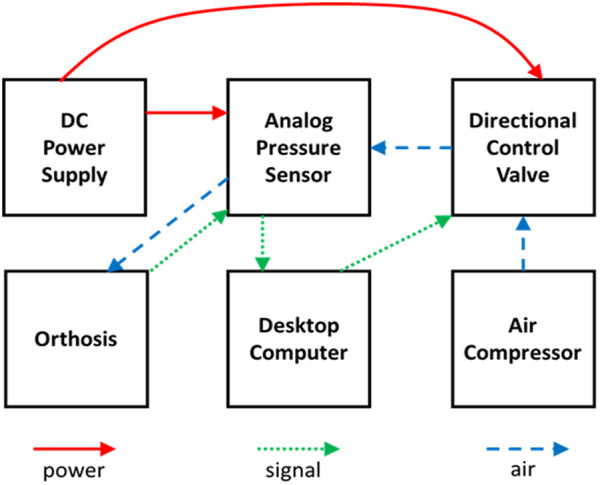
Experimental setup. This block diagram shows the experimental setup. The arrows indicate flow of power (red, solid), signal (green, dotted), and air (blue, dashed) for device inflation.

At present, the orthosis prototype is actuated via an air compressor, valves powered through a DC power supply and controlled through a computer interface. This is an experimental platform for pressure control and sensing developed for use in the subject testing. A diagram of the setup is shown in Figure 4. A pressure supply air line was connected to a Festo proportional directional control valve, powered by a 24 V DC supply and controlled by Simulink Real-Time via a National Instruments data acquisition card. The valve was connected to an analog pressure sensor (Festo SDE-16-10 V/20 mA), which attached to the orthosis via ¼” pneumatic tubing. The valve is a three-position, continuously controllable valve, where the first position connects the orthosis to supply pressure, the middle is flow blocked, and the third is exhaust. Being a proportional valve, supply pressure could be slowly admitted into the orthosis by controlling the valve orifice. The valve was opened, and pressure was slowly, manually increased to inflate the orthosis to the desired grip force, and that value was then set as the desired peak pressure for open loop operation.

**Figure 5.**
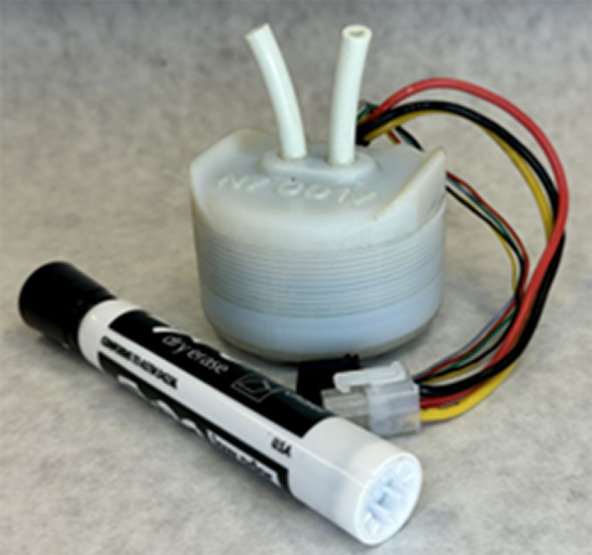
Bidirectional gerotor pump (Gallentine and Barth, 2023). The pump is shown with a dry erase marker for size scale.

Eventually, the system is intended to be fully portable, with an on-board power and control system consisting of batteries and a bidirectional pump mounted on the upper arm, similar in size and weight to a typical blood pressure cuff. The design for this addition is already in development, and the current iteration, shown in Figure 5, fits within a 2.25-inch cube, weighs 360 g, and has a manufacturing cost of 18 USD (Gallentine and Barth, 2023).

## 4. Testing and results

### 4.1. Experimental protocol

In order to assess the feasibility of the prototype in meeting the primary design constraints and facilitating gross motor functions for basic daily activities, the prototype was tested on a stroke survivor. This test subject was recruited from a brain injury rehabilitation clinic through the recommendation of an occupational therapist. The subject was a 56-year-old male, 10 months post-stroke, with a completely flaccid upper arm until the shoulder joint (no volitional control of hand, wrist, or elbow). Details about the subject are shown in Table 3. The study was approved by the Vanderbilt University Institutional Review Board, study number 221203, and all subjects provided informed consent to participate.

**Table 3.** Test subject information

Injury	Stroke
Months post-injury	10
Gender	Male
Age (years)	56
Tone level	Flaccid until shoulder

The subject came with his caregiver to the laboratory to test the orthosis. Three types of tests were performed: donning and doffing, grasping/ADLs, and cyclic exercise. The sequence of events for each session was as follows, with performance metrics shown in parentheses:

1. Donning and doffing tests (time)
2. Grasping common objects tests (pressure)
3. ADL tests (time, performance quality)
4. Cyclic exercise tests (pressure vs. time)
5. Interview subject for feedback

When deciding the best protocol for orthosis assessment, the authors considered several standardized hand function tests: the Functional Independence Measure, as well as the Sollerman, Jamar, Jebson-Taylor, and Toronto Hand Function Tests. Standardized assessments are important in a clinical setting when statistical results from large sample populations are desired; however, they are not commonly used in engineering research laboratories for testing prototypes due to their broadness. Most of these tests incorporate a wide range of activities and grasps that are beyond the scope of this work. Therefore, in keeping with this prototype’s goals of gross motor assistance, the authors developed a protocol most similar to a modified version of the Toronto Hand Function Test.

### 4.2. Donning and doffing

The donning and doffing tests were performed with the patient seated at a table. First, the patient’s caregiver was timed to determine how long it took her to put the orthosis on the patient securely and then remove it. Due to the patient’s inability to move his elbow and therefore position his arm, he was unable to don the device himself, even when his arm was initially set on the table for him, as his arm kept sliding off, and then, he could not reach his hand. However, he was able to remove the orthosis independently, so this activity was timed as well.

Both the caregiver and the subject were easily able to remove the orthosis from the subject’s hand. The subject could not don the orthosis unassisted because of his lack of arm control and therefore inability to position his arm (it kept sliding off the table as he moved), so the only don time shown is by his caregiver. The donning and doffing times are listed in Table 4.

**Table 4.** Donning and doffing times

Subject	Don time (s)	Doff time (s)
Subject (assisted)	42.4	8.3
Subject (unassisted)	n/a	13.3

### 4.3. Grasping and ADL testing

**Figure 6.**
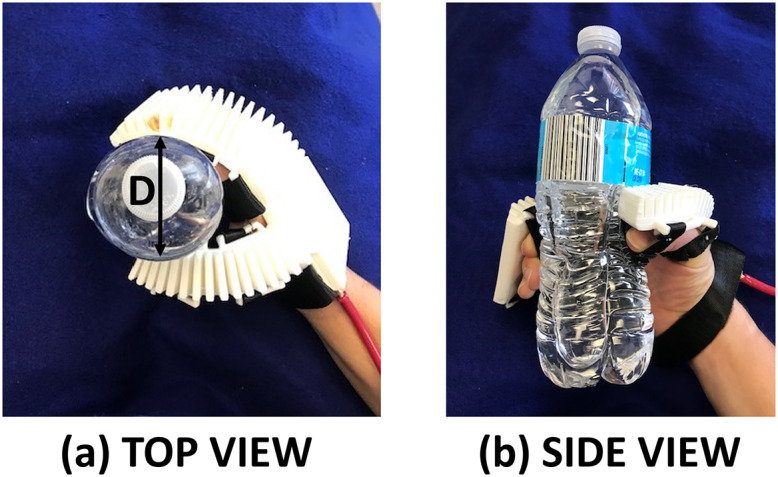
Example ADL: grasping water bottle. These images show a hand gripping a water bottle with the help of the orthosis. The left photograph (a) shows the top view, with grip dimension D, while the right photograph (b) is the side view.

The grasping tests were performed with common household objects of various sizes and compliances: a cell phone, a banana, a disposable water bottle, and a screwdriver. The subject was instructed to use his healthy hand to position an object in his affected hand, and the orthosis inflation pressure was manually increased by the researcher until the participant confirmed that a stable grip was achieved. The subject then shook his arm to verify stability. An example photograph of holding a water bottle is shown in Figure 6.

Two bimanual ADL tests were performed, using the orthosis-clad affected hand to stabilize an object while the unaffected hand performed a task. The inflation pressures for these tasks were set based on those determined from the grasping test. The first ADL test involved opening and closing water bottles. The subject was seated at a table with five disposable water bottles approximately 60% full. He was instructed to pick up a water bottle, stabilize it with his affected hand, unscrew the cap, and replace the bottle. This sequence was repeated for all five water bottles. The test was timed, and any spillage was noted. Then, the entire test was repeated for re-capping the water bottles, with time and spillage again noted.

**Figure 7.**
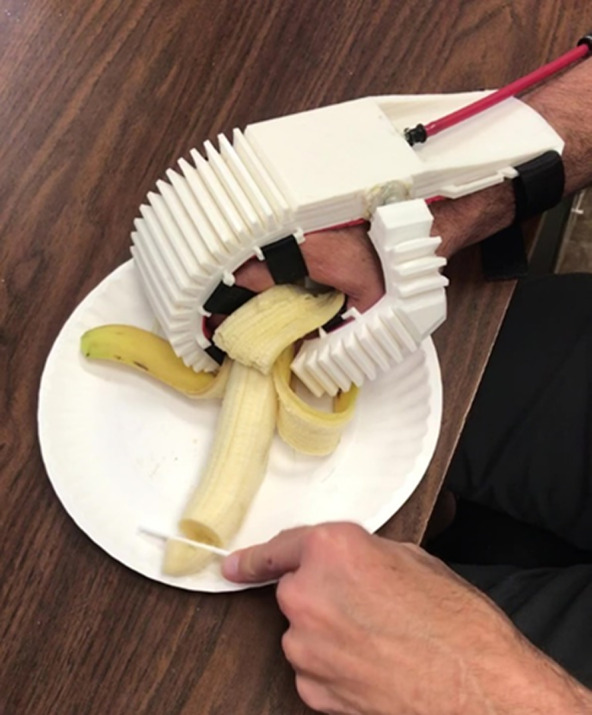
Example ADL: slicing banana. This photograph shows the subject stabilizing and slicing a banana with the help of the orthosis.

The second ADL test was holding and slicing fruit. A banana and plastic knife were used for safety reasons, but the test simulates the same operation as using a traditional knife on any cylindrical fruit, vegetable, or similar object with soft composition. (This is not meant to extend to spherically shaped fruits and vegetables such as apples and onions due to the different grasp type required and lower surface area available for stability.) For this test, the subject gripped the banana in his orthosis-clad affected hand and held it horizontally on a paper plate. He then used his unaffected hand to cut seven slices off the banana. The test was timed, and the thickness and uniformity of the slices measured. A photograph of this test is shown in Figure 7.

The pressure required to stably grasp each object is shown in Table 5 along with *D*, the “grip dimension” (see Figure 6), the farthest distance between points of contact of the hand on the object. (For cylindrical objects, this is the diameter of the object, and for the cell phone, it is the width of the phone.) All the objects were held stably using the orthosis, as none of them slipped when the subject shook his arm. Without the orthosis, the subject was not able to hold anything with his affected hand.

**Table 5.** Grasping dimensions and pressures

Object	Grip dimension (cm)	Actuator pressure (psi)
Banana	3.3	21
Water bottle (compressible)	6	23
Cell phone	7	30
Screwdriver	3	31

**Figure 8.**
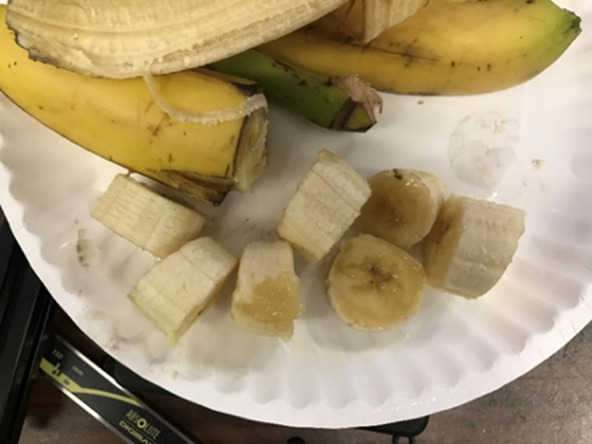
Sliced banana. This photograph shows the seven banana slices uniformly cut by Subject 1 during his test.

Both the water bottle and banana slicing tests were successfully completed by the subject. Video footage of the testing is available here. The five water bottles were opened in a total of 38 s (7.6 s per bottle) and closed in a total of 33 s (6.6 s per bottle), compared to not being able to grasp, open, or close the bottles at all without the orthosis. No water was spilled in either case. For the banana slicing test, the subject was able to hold and slice the banana with the orthosis, while without it he could do neither. The subject was able to hold the banana without slipping and slice seven pieces in 10 s total, all quite uniform at about 1.8 cm thickness except for one smaller slice. A photograph of the sliced banana is shown in Figure 8.

The grip force of the orthosis was tested separately using a dynamometer. The soft actuator was positioned around the dynamometer handle and manually inflated as grip force was measured. The grip force test was unable to be performed with the patient due to his hand flaccidity (could not grip anything unassisted), and after donning the orthosis, his hand was too large to fit in the dynamometer handle. The maximum grip force achievable by the orthosis was 48 N at 65 psi (448 N). This exceeds both the target force of 40 N and the pressure range of 20–30 psi required for stable grasping observed during the experiments.

### 4.4. Cyclic pressure testing

**Figure 9.**
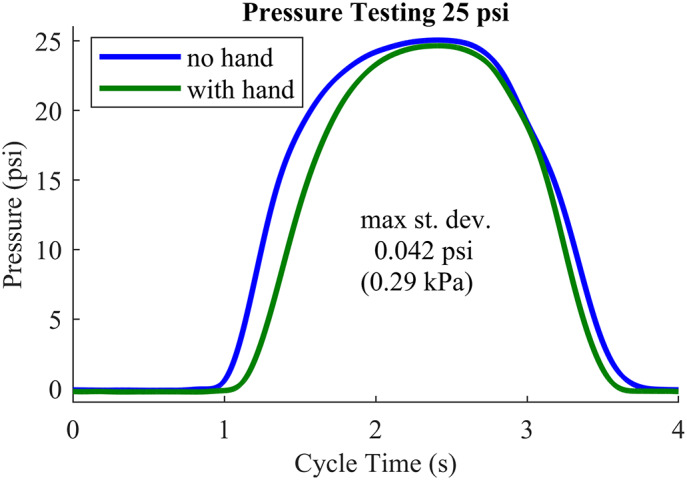
Cyclic exercise pressure testing. Average pressure over 10 cycles at 25 psig (172 kPa). The blue solid curve is the device pressure without human interaction, and the green solid curve is the average device pressure when worn by the subject. Curves for 20 and 30 psig look similar.

The cyclic exercise tests were performed at three different peak pressures: 20, 25, and 30 psig (138, 172, and 276 kPa). These pressures were selected based on the range required for gripping common household objects and the fact that 30 psig completely closed the subject’s hand (fingertips touching palm). The subject wore the orthosis, which was automatically inflated and deflated by continuously varying the valve orifice with a sinusoidal cycle with a 4-second period, for one minute. This test was performed three times, once for each peak pressure, with one minute of rest in between. During the testing, the subject was carefully observed and communicated with to ensure his hand was not in pain. The cyclic pressure test was also performed with the orthosis on its own (not worn by a human) to collect baseline data for comparison. Pressure history averages over 10 stretching cycles are shown in Figure 9. The testing showed a high degree of repeatability at 20, 25, and 30 psig maximum inflation.

### 4.5. Subject feedback

Following the testing, the subject was informally interviewed to determine his perspective on the orthosis and testing session. The researchers wished to learn the subject’s opinion of the device and suggestions for device or protocol improvement.

The subject found the orthosis to be comfortable due to its foam padding and flexible structure yet stable straps and said “it feels just right, it works, I really like that.” He further appreciated unobstructed contact sensation between his hand and the objects grasped. The caregiver found the Velcro straps easy to use for donning, doffing, and adjusting the orthosis for the patient.

## 5. Hand–actuator kinematics

It would be useful to characterize the soft actuator’s bending response to pressure, both with and without a hand wearing the orthosis (“loaded” and “unloaded,” respectively), in order to better understand the hand–actuator interactions. This provides a qualitative measure of both the actuator’s consistency and the resistance caused by the hand, and a way to predict the joint angles of the user’s fingers as the actuator curls.

**Figure 10.**
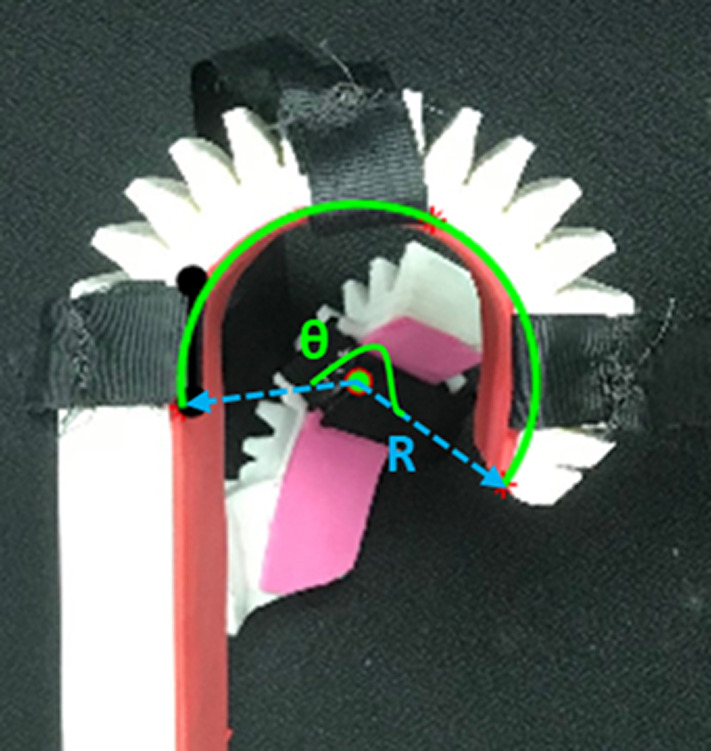
Soft actuator at 20 psig (138 kPa). This image shows a side view of the unworn pressurized orthosis with radius of curvature R and angle of curvature 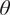 _._

The experiment began with pressurizing the soft actuator from zero to 40 psi (276 kPa) in increments of 5 psi (40 kPa) and photographing a sagittal view at each step. This was done for three scenarios: (1) the actuator unloaded, (2) the orthosis worn by a small, 20th percentile female hand, and (3) the orthosis worn by a large, 90th percentile male hand. The photographs were post-processed in MATLAB to measure the radius and angle of curvature at each pressure, as shown in Figure 10.

These points were then plotted, and best-fit curves were found to characterize the actuator’s performance. The types of fits were selected based on the shapes of the plots: radius of curvature followed a negative exponential relationship and angle of curvature followed a linear relationship for the unloaded actuator and a second-order relationship for the loaded actuator.

**Figure 11.**
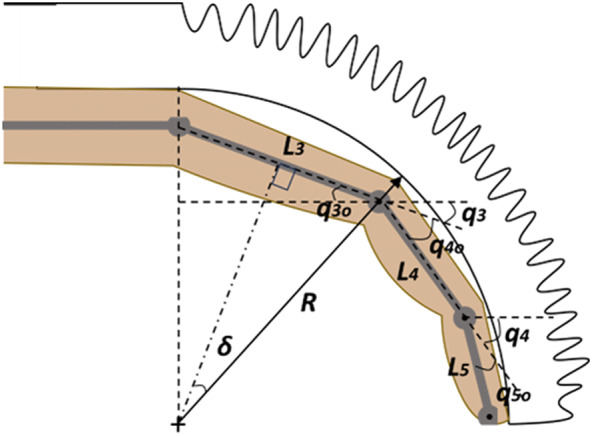
Kinematic diagram of soft actuator with hand. This diagram shows the three segments of the finger connected by the MCP (proximal, q3), PIP (middle, q4), and DIP (distal, q5) joints.

After determining the best-fit relationships for pressure and curvature, the hand of the wearer was approximated as rigid links connected by revolute joints. As the fingers of the hand are the only part that the orthosis bends, only three joints were of interest: MCP, PIP, and DIP. Assuming a constant radius of curvature for the actuator, the finger was assumed to touch the inside of the curve at each joint. Using the lengths of the finger segments and newly obtained relationship between the actuator’s radius of curvature and internal pressure, the angles of the three joints can be predicted via kinematics, as shown in Figure 11.

Labeled on the diagram are joint angles *q*, link lengths *L*, and radius of curvature *R.* The subscript *i* represents the joint number and subsequent link to which it is attached. From most distal to most proximal, these are 5/DIP, 4/PIP, and 3/MCP. Each joint angle can be calculated as follows:
(5.1)

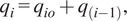

where
(5.2)

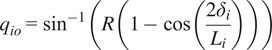

and
(5.3)

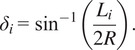



**Figure 12.**
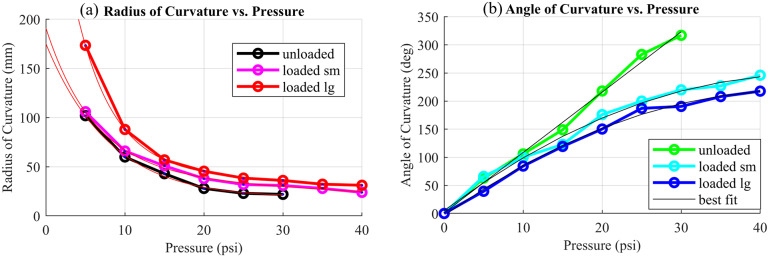
Radius and angle of curvature. (a) shows the radius of curvature data for the unloaded, loaded small subject, and loaded large subject in black, magenta, and red, respectively. Best-fit curves are shown in red. (b) shows the angle of orthosis curvature for the unloaded, loaded small subject, and loaded large subject in green, cyan, and blue, respectively. Best-fit curves are shown in black.

In order to test these relationships and the kinematic model of the hand, the same experiment was repeated at five different pressures in between the previously collected points for the loaded actuator with both subjects. The data from these experiments were processed in MATLAB and compared to the predicted results. Figure 12a,b shows the best-fit curves for the radius and angle of curvature, respectively, for the actuator unloaded, loaded with the small hand, and loaded with the large hand.

The relationship between pressure and angle of curvature for the soft actuator was found to be linear for the unloaded case ( 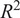 = 0.997) and second order for both loaded cases ( 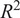 = 0.947 small subject, 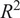 = 0.946 large subject). The formula for the relationship is
(5.4)

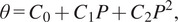

 where 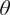 is angle of curvature, *P* is pressure, and *C* is the coefficients. The values determined for each coefficient are shown in Table 6, for pressure in psi and angle in degrees:

**Table 6.** Angle of curvature coefficients

Condition	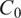 (deg)	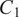 (deg/psi)	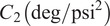
Unloaded	1.25	10.74	0
Small subject	5.37	10.50	−0.11
Large subject	−3.56	10.00	−0.11

The relationship between pressure and radius of curvature was found to be a two-term exponential relationship for all cases. The formula for the relationship is
(5.5)

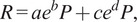

 where *R* is radius of curvature in mm, *P* is pressure in psi, and *a, b, c*, and *d* are coefficients. The values determined for the coefficients are shown in Table 7.

**Table 7.** Radius of curvature coefficients

Condition	a (mm)	b ( 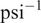 )	c (mm)	d ( 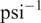 )
Unloaded	165.7	−0.119	9.44	0.0194
Small subject	143.7	−0.168	47.4	−0.0165
Large subject	367.6	−0.216	52.3	−0.0133

**Figure 13.**
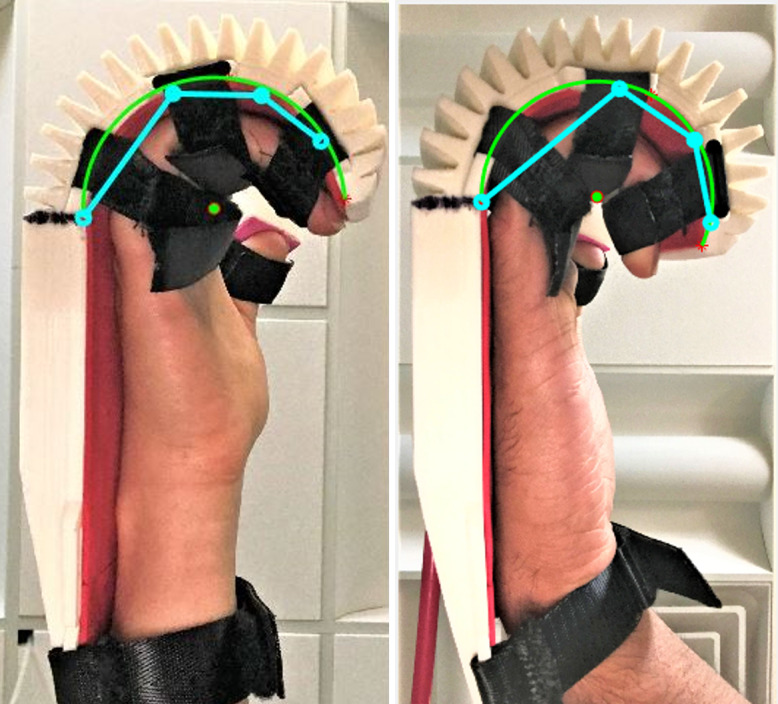
Loaded soft actuator under pressure. This image shows a side view of the orthosis pressurized to 20 psig (left) while worn by the small subject and 32.5 psig (right) while worn by the large subject. Overlaid on the photograph are the actuator curvatures in green, with the center of curvatures as green dots, and the predicted finger profiles in cyan.

**Figure 14.**
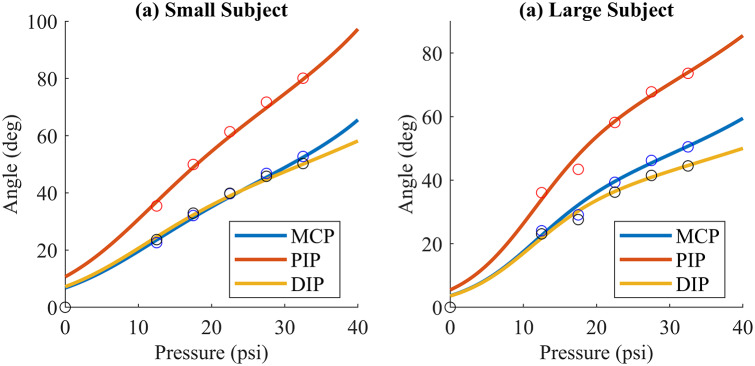
Actual versus predicted joint angles. The actual joint angles (dots) and predicted joint angles (solid curves) are shown for the small (top) and large (bottom) subjects. The blue, red, and yellow curves represent joints MCP, PIP, and DIP, respectively.

Figure 13 shows the hand of each subject wearing the pressurized orthosis, with the actuator curvature and kinematic diagram of the fingers overlaid. One can see here that the actual positions of the fingers reflect the predicted kinematics. Plots of the actual versus predicted joint angles are shown in Figure 14a,b. This modeling provides a therapist with the inflation pressure required for any desired joint angle at any of the three joints, provided that a model of this form has been developed for the patient in question.

## 6. Discussion

The orthosis prototype is flexible, comfortable, sturdy, and nears or exceeds all primary design criteria. The primary significance of the testing results is that the subject could hold objects and perform daily activities using the orthosis, while he was otherwise completely unable to use his affected hand. The prototype weighs 276 g for the 50th percentile male size and within the typical range of 120–400 g reported in literature (Kadowaki et al., 2011; Polygerinos et al., 2015; Gasser et al., 2017; Saharan et al., 2017; Yap et al., 2017b; J. Wang et al., 2019; Yoo et al., 2019; Hong et al., 2020; L. Wang et al., 2020; Yurkewich et al., 2020; Shi et al., 2021; Tran et al., 2022). Compared to existing commercial technology, which is in many ways more refined and advanced than research prototypes, the orthosis offers the benefits of being lighter, cheaper, softer, and easier to don than most. The fabrication cost for the wearable device is under 30 USD (excluding the off-board power and control system). Furthermore, the device size is customizable based on patient hand length and width, requiring less than a minute to regenerate and download new part files for 3D printing. Both the test subject, his caregiver, and the occupational therapist approved of the device’s fit and functionality.

While the donning and doffing tests were both speedy and successful for the caregiver (the don time of 42 s and doff time of 8 s were well below the 60-s target) and significantly faster than those in published literature, the subject himself was unable to don the orthosis independently. This was due to a lack of volitional control and tone throughout his arm, which hung limply by his side except for some shoulder movement. Therefore, even when his arm was placed on the table for him, he was unable to use his unaffected hand to simultaneously position the orthosis on his affected hand and fasten the straps. Whenever he tried to improve his position, the motion of his body caused his arm to slide off the table. The authors speculate that if the subject had control of his elbow (as some other patients do) and could position his arm, the procedure could have been more successful. He was able to independently remove the orthosis once donned, as this operation did not require specific hand position, and did so in 13 s, more slowly than the caregiver but still well below the 60-s threshold. The next fastest time for donning a hand orthosis reported in literature is 3 min (Yurkewich et al., 2020), and the subjects in that study required assistance.

The power grasping test was successful, as the subject was able to securely hold each of the four objects with the orthosis even when shaking his arm (without the orthosis, his affected hand could not function at all). Pressures were all between approximately 20–30 psi, but the pressure required for stability did not strictly correspond to object size. This is likely due to the different shapes, stiffnesses, and friction coefficients of the objects, and the two softer objects (water bottle and banana) required lower pressures for stability than the rigid objects (screwdriver and cell phone). The maximum grip force of the prototype, 48 N at 65 psi, exceeds both the pressures required from the grasping experiments and the baseline grip force of 40 N required for ADLs (Riddle et al., 2018). Furthermore, it exceeds the grip forces of 25–38 N from the strongest soft robotics research prototypes reported in Rieger and Desai (2022), Suulker et al. (2022), Yap et al. (2017b), and Zhou et al. (2019) as well as those in Hong et al. (2020), Kadowaki et al. (2011), Kladovasilakis et al. (2022), Li et al. (2023), Polygerinos et al. (2015), Tran et al. (2022), J. Wang et al. (2019), L. Wang et al. (2020), Yap et al. (2017a), Yurkewich et al. (2020), and Zhao et al. (2016).

The subject performed the ADL tests with the water bottles (vertical grip) and banana (horizontal grip) quickly, accurately, and stably. He picked up, opened, and replaced all five water bottles in under 40 s, less than 8 s per bottle, and closed them even more quickly, without spilling any water in either case. The disposable water bottles were only filled to 60% capacity with the goal of reducing spillage, so it is possible that fuller bottles would have been messier. The subject was able to stabilize the banana with his affected hand and slice uniform 1.8-cm-thick pieces from it with his unaffected hand. Neither the banana nor the plastic knife slipped during the procedure. While a banana is softer to cut than other fruits and vegetables, the process is the same. The researchers anticipate this performance could extend to other cylindrical foods such as cucumbers, zucchini, and carrots, but it is unknown how spherically shaped fruits and vegetables such as apples and onions would compare due to the different grasp type required and lower surface area available for stability.

The orthosis performed uniformly and consistently during the cyclic pressure testing for both the subject and the “unloaded” tests. One can see that the pressure is essentially zero until the device has reached a threshold of inflation volume, at which point the pressure rapidly increases, peaks, and then drops off as the air exits. This performance is so consistent that the small standard deviations are nearly invisible on the plot. The pressure increases sooner and drops later for the “unloaded” device than when worn by the subject, with a flatter peak. This indicates that the subject’s hand, although flaccid, caused some level of load against the orthosis. An interesting behavior observed during this test was the subject mirroring hand motion (without any suggestion from the authors). As the orthosis guided the subject’s affected hand to open and close, he consciously mirrored this activity with his unaffected hand. When asked about this behavior, the subject explained that the exercise was an attempt to retrain his mind–body connection to improve the function of his affected hand, indicating that he saw the device as a useful therapy tool.

The test subject’s feedback was positive. He was thrilled to be able to use his affected hand to perform activities he had not done in almost a year, since his stroke. He kept exclaiming happily at each successful activity. The subject was pleased with the useful applications tested and began speculating on other activities for which the orthosis would help, remarking that “the more you do it, the easier it gets.” An added benefit to the comfort and functionality of the device was the contact sensation maintained between his hand and the objects grasped. Additionally, both the subject and caregiver approved of the Velcro straps for ease of donning and doffing.

The occupational therapist sanctioned the orthosis design, citing in particular the wrist support, thumb strap position, and grasping form. She found it to be easy to put on patients and approved of the comfortable padding to prevent hand abrasion, especially because some patients lose sensation in their affected limb and are less attentive to sores developing.

While limitations regarding the ADL tests have been previously discussed, the study has some other limitations as well. Primarily, this study used a small sample size (n = 1), so the results cannot be generalized to the entire stroke survivor population. It is also noteworthy that the full benefits of this device are limited to individuals with a functioning shoulder and elbow on the affected side, as the subject was not able to don the orthosis himself or orient his affected arm due to flaccidity. The orthosis in its current form has some limitations as well, considering that it is not self-contained (the power source and controls are still off-board) and requires more development before it could be used by patients at home or independently.

From the hand–actuator kinematics plots, one can see that it is possible to relate radius and angle of curvature to pressure using exponential and linear or second-order polynomials, respectively. It is interesting that the relationship between pressure and angle of curvature is linear without a load and becomes nonlinear when the orthosis contains a hand. This indicates that the hand, although flaccid, applies a small nonlinear load to the orthosis. Also noteworthy is that this resistance is proportional to the size of the hand, as evidenced by the different curves for the small and large hands. The experimental testing yielded radius and angle of curvature and joint angles close to the predicted values in all cases. The actual joint angles fell close to the predicted values, as shown by the plots, and observing the photographs with overlaid kinematic diagrams passes a visual reality check. In the photos, one can see some minor differences between the fingers and kinematic diagrams. These are due in part to the approximation of the fingers as having uniform length equal to the index finger, whereas in reality, the middle finger of the hand is longer and the pinky is shorter, and furthermore, the fingers have thickness unaccounted for in the simplified model.

The WHO prototype performed reliably and neared or exceeded all design and functionality targets. The design is sturdy and reliable. The flexibility and motion of the device improve upon the static splints currently in use, and the wrist support, grip strength, and ease of donning and doffing improve upon other research prototypes. Furthermore, both the test subject and the occupational therapist expressed approval for the device design and performance. Although this study focused on stroke survivors as the initial target demographic, the orthosis has the potential to be utilized by other neurologically impaired patients as well.

## 7. Conclusion

The design of the orthosis prototype is robust and effective, solving issues with existing technology and current research prototypes. The primary design criteria of easy to don and doff, soft, inexpensive, assistive grasping, and therapeutic exercise have been met. The device design can be quickly and easily custom-sized based on two parameters, and it is the first powered soft robotic hand orthosis to have this benefit, as well as to preserve direct contact between the palmar side of the hand and the object grasped. The initial evaluation of functionality was successful and sets the stage for further prototype development (portability and controls) and evaluation of long-term effectiveness.

## Supporting information

Ledoux and Barth supplementary material Ledoux and Barth supplementary material

## Data Availability

Derived data supporting the findings of this study are available from the corresponding author upon reasonable request. Certain data such as subject names and contact information will not be available due to privacy requirements. The anatomical data used for the custom sizing modeling are available in Gordon et al. (1989).

## Supplementary material

The supplementary material for this article can be found at http://doi.org/10.1017/wtc.2024.18.
