# Supplementary material for: Design, modeling, and preliminary evaluation of a 3D-printed wrist–hand grasping orthosis for stroke survivors
Source: Wearable Technol. 2024 Nov 8;5:e12. doi: 10.1017/wtc.2024.18 (PMC11579884; doi:10.1017/wtc.2024.18)
Supplement: Ledoux and Barth supplementary material [file S2631717624000185sup001.pdf]

Customer Discovery Questions

These questions were used as talking points in customer discovery conversations. Due to the informal nature of the discussions, questions could be removed or added as the conversation evolved.

General

What physical challenges do stroke survivors face? How often? Are they a big deal?

What are the three hardest things to do?

Is there anything I haven't asked that I should know?

Any contact referrals?

Survivors

How long has it been since you have a stroke?

What physical challenges do you face? How often? Are they a big deal?

What are the three hardest things to do?

Do you live on your own or with assistance?

Have you or do you use any assistive devices?

If money weren't an obstacle, would you do/want anything differently?

Caregivers

How long has it been since your spouse/relative/dependent had a stroke?

What physical challenges does he/she face? How often? Are they a big deal?

What are the three hardest things for him/her to do?

How much assistance (magnitude, frequency) do you provide?

Does he/she use any assistive devices?

If money weren't an obstacle, would you do/want anything differently?

Providers (Therapists, Doctors, Nurses)

What population do you work with, in what capacity?

What physical challenges do stroke victims face? How often? Are they a big deal?

What are the three hardest things for patients to do?

Do patients use any assistive devices?

What factors affect recovery?

Is there anything you think all stroke victims should have access to?
